# Mechanism by *Sambucus nigra* Extract Improves Bone Mineral Density in Experimental Diabetes

**DOI:** 10.1155/2012/848269

**Published:** 2012-09-13

**Authors:** Laurentiu Badescu, Oana Badulescu, Magda Badescu, Manuela Ciocoiu

**Affiliations:** ^1^Department of Cellular and Molecular Biology, University of Medicine and Pharmacy “Grigore T. Popa” Iasi, 16 Universitatii Street, 700115 Iasi, Romania; ^2^Department of Pathophysiology, University of Medicine and Pharmacy “Grigore T. Popa” Iasi, 16 Universitatii Street, 700115 Iasi, Romania

## Abstract

The effects of polyphenols extracted from *Sambucus nigra* fruit were studied in streptozotocin- (STZ-) induced hyperglycemic rats to evaluate its possible antioxidant, anti-inflammatory, antiglycosylation activity, and antiosteoporosis effects in diabetes. DEXA bone mineral density tests were performed in order to determine bone mineral density (BMD), bone mineral content (BMC), and fat (%Fat) in control and diabetic animals, before and after polyphenol delivery. As compared to the normoglycemic group, the rats treated with STZ (60 mg/kg body weight) revealed a significant malondialdehyde (MDA) increase, as an index of the lipid peroxidation level, by 69%, while the total antioxidant activity (TAS) dropped by 36%, with a consistently significant decrease (*P* < 0.05) in the activity of superoxide dismutase (SOD) and glutathione peroxidase (GPX). Also, the treatment of rats with STZ revealed a significant increase of IL-6, glycosylated haemoglobin (HbA_1c_), and osteopenia detected by DEXA bone mineral density tests. The recorded results highlight a significant improvement (*P* < 0.001) in the antioxidative capacity of the serum in diabetic rats treated with natural polyphenols, bringing back to normal the concentration of reduced glutathione (GSH), as well as an important decrease in the serum concentration of MDA, with improved osteoporosis status. Knowing the effects of polyphenols could lead to the use of the polyphenolic extract of *Sambucus nigra* as a dietary supplement in diabetic osteoporosis.

## 1. Introduction

Observational studies and animal models suggest that decreased bone strength in diabetes may contribute to fracture risk but this remains a controversial issue [1]. Diabetes can impact bone through multiple pathways including changes in insulin levels, higher concentrations of advanced glycation end products in collagen, hypercalciuria associated with glycosuria, oxidative stress, lower insulin-like growth factor-I, and inflammation [2]. Epidemiologic studies suggest a protective role of dietary flavonoids against coronary heart disease [3]. Duration of diabetes seems to play a key role given the lower bone mineral density found among patients who have had diabetes for over 5 years [4]. In our study, a 12-week hyperglycemia in rats is equivalent to a 6-7-year diabetes mellitus (DM) in humans. A better understanding of these mechanisms may help implement fracture prevention measures in the growing population of adult diabetics.

It is known that natural polyphenols possess physical and chemical properties that contribute to the proper and efficient protection against oxidation of important biomolecules such as lipids, proteins, and nucleic acids [5]. Flavonoids are a major class of active antioxidant principles.

The polyphenolic extract from the isolated and purified vegetable material, which is the mature *Sambucus nigra* (*Caprifoliaceae*, elderberry) fruit, can constitute an important source of antioxidants. DEXA bone mineral density tests were performed in order to determine bone mineral density (BMD), bone mineral content (BMC), and fat (%Fat) in control and diabetic animals, before and after polyphenol delivery. Our goal is to develop a new alternative treatment that includes a dietary supplement designed to reduce bone loss in diabetic patients.

## 2. Material and Methods

### 2.1. Preparation of Extract and Chemical Determinations

Elder fruits (*Sambucus nigra* L.) were washed and shade dried. Dried and powdered fruits (50 g) were extracted with 2 × 250 mL acidulated methanol (0.5% HCl) using a magnetic stirrer, each time for one hour. The amount of vegetable extract obtained from *Sambucus nigra* fruit was 42.31 g/100 g dry vegetable material.

The total phenolic content in elder fruit extract was determined by the Singleton and Rossi method. The amount of total phenolic content was expressed as gallic acid equivalents (GAE)/100 g extract. The absorbencies of all the solutions were determined by using a V-550 Able Jasco UV-VIS spectrophotometer. The result is the mean of triplicates ± standard deviation. The content in total polyphenols from the *Sambucus nigra* extract was 7,63 ± 0,12 mg GAE/100 g extract.

Recent studies have revealed a total phenolic content of 97.26 ± 17.04 mg GAE/100 g in dried Coriander seeds (*Coriandrum sativum*), of 63.51 ± 3.67 mg GAE/100 g in garlic (*Allium sativum*), and of 16.20 ± 0.96 mg GAE/100 g in potatoes (*Solanum tuberosum*) [6].

The dry polyphenol extract was diluted in DMSO, 100 mL polyphenolic solution containing 840 mg natural polyphenols, 95 mL distilled water, and 5 mL DMSO. After repeated testing, it was found that the dose of polyphenols extracted from the fruits of *Sambucus nigra* to be administered as enteral solution (by tube feeding) is 0.040 g/Kg body every two days. The experiment used active therapeutic doses, well-determined fractions of DL50 on an experimental model of diabetes mellitus.

### 2.2. Experimental Model

The research was carried out on Wistar white male rats, aged 28 weeks at the beginning of the study, weighing 250–280 g on the average, which were divided into four 12-rat groups, namely, W Group: control, normal animals, that were not given natural polyphenols; DM Group: rats with diabetes caused by the rapid intraperitoneal injection of a single dose of streptozotocin (STZ); PGroup: rats who were administered polyphenolic extract for a period of 16 weeks; DM + P Group: rats who were administered a polyphenolic extract for 3 weeks before and 13 weeks after diabetes mellitus induction.

Diabetes was induced by STZ [2-deoxi-2(3-metil-nitrozo-ureido)-p-glucopiranoza] (cytotoxical antibiotic synthesized of *Streptomyces achromonogenes*) obtained from SIGMA S-0130, Batch 31K1379, and delivered in a single dose of 60 mg/Kg body mass, as 1% intraperitoneal (i.p.) solution, after 18 hours of fasting. Streptozotocin delivery causes insulin-deficiency diabetes through selective endocrino-chemical pancreatectomy, destroying only the cells of Langerhans' islets [7]. The onset of permanent diabetic hyperglycemia occurs after 24–48 hours from the diabetogenic injection and it may either be definitive or regress spontaneously after a few days, depending on the animal's diet and specific characteristics. 

The animals were kept in normal microclimate conditions. The clinical state of the animals was observed daily, their water and food ingestion, diuresis, glycosuria, and the possible presence of ketone bodies. The animals were fed using a daily intake calculated according to the standard norms for the species. The diet consisted of carbohydrates 59.12%, raw proteins 21.10%, raw lipids 5.08%, raw fibers 4%, minerals 5.14%, humidity of 7.98%.

The experimental study fulfils all the requirements of the guide regarding the use of laboratory animals and biological preparations issued by the International Society of Pain Study (IASP) and the European Council Committee (86/609/EEC). This study was approved by the Laboratory Animal Care Committee of “Gr. T. Popa” University of Medicine and Pharmacy and the rats were kept in accordance with the general guidelines for the care and use of laboratory animals recommended by the Council of European Communities. At the end of the experiment, the 44-week-old animals were killed by cardiac puncture under ketamine anaesthesia (100 mg/kg body weight). Blood samples were collected using sodium citrate as anticoagulant buffer, at a 9:1 blood/citrate ratio, or without anticoagulant. Aliquots of plasma and serum were frozen and kept at −80°C for later analysis.

### 2.3. Biochemical Methodology

Fasting blood glucose was measured by routine autoanalyzer methods (Synchron CX 7, Beckman), using dedicated kits. TAS was measured in heparinized plasma samples by the method of Miller et al., using the Trolox equivalent antioxidant capacity (TEAC) assay (Randox Laboratories, San Francisco, CA). Both water- and lipid-soluble antioxidants contained in the biological sample under investigation inhibit the 2,2′-azinobis (3-ethylbenzothiazoline 6-sulfonate) radical cation production. Radical cation production was measured by spectrophotometry. 

The overall blood glutathione peroxidase (GPX) activity was determined using the method of Paglia and Valentine. Kits for TAS and GPX activity analysis were purchased from Randox (United Kingdom). Blood superoxide-dismutase (SOD) was determined by an adapted method from Minami.

Malondialdehyde (MDA) was determined by measuring thiobarbituric reactive species using the method of Ruiz-Larrea et al. [8] in which the thiobarbituric acid reactive substances react with thiobarbituric acid to produce a red-colored complex having peak absorbance at 532 nm. 

Bio-Rad's Micromat II Hemoglobin Instrument was used for HbA1c testing, as it provides results in less than 5 minutes. Based on borate affinity chromatography, there is no interference from abnormal haemoglobins, which makes it very reliable. 

IL-6 serum levels were measured by enzyme-linked immunosorbent assay.

The *DEXA* bone mineral density test was performed in an Endocrinology Clinic on HOLOGIC 100 equipment, using special software for small animals, Dynamic evolution of bone mineral density was analyzed, namely the fat percentage in the entire body.

### 2.4. Statistical Data Interpretation

Data were expressed as means ± standard deviation (SD). Statistical significance was determined by variance analysis and one-way ANOVA followed by a post hoc Tukey's test using Statistical Software Package SPSS, version 13 (SPSS Incorporation, Chicago, IL, USA). Unpaired Student's *t*-tests were performed to determine whether there were significant (*P* < 0.05) differences between groups. The statistical interpretation of the data considered the corresponding differences for a significance threshold: *P* > 0.05 statistically insignificant; *P* < 0.01 strong statistical significance; *P* < 0.001 very strong statistical significance.

## 3. Results

Table 1 shows the dynamic evolution of glycaemia in the diabetic rats under polyphenolic protection as compared to the unprotected diabetic rats.

10 weeks after the beginning of the experiment, the hyperglycemia of the DM + P group compared with the DM group was insignificantly low (*P* > 0.05). Thus, polyphenol delivery did not provide protection against disease onset, yet it reduced glycaemia evolution insignificantly. Our study revealed significant hyperglycemia reduction in the DM+P group compared with the DM group, 16 weeks after the beginning of the experiment (*P* < 0.01).

Serum TAS, GPX, and SOD activities were significantly decreased in the DM group. There is evidence to suggest that oxidative damage is increased in diabetes. Antioxidant activity was improved by polyphenol addition in comparison with the unsupplemented groups. Also, the MDA values revealed the antioxidant effect of polyphenols on diabetic animals (Table 2). There were associations between TAS and HbA1c (*r* = −0.43; *P* = 0.0026).

*Bone mineral density* tests were performed on laboratory animals at the beginning and at the end of the experiment, respectively. Three parameters were analyzed: BMD (bone mineral density expressed in g/cm^2^); BMC (bone mineral content expressed in g), and Body fat (%Fat).

The BMD evolution compared with the area measured in control males throughout the experiment was not statistically significant in any of the bone mineral density tests: overall *χ* ^2^ = 0.03; GL = 1; *P* = 0.85; spine *χ* ^2^ = 0.30; GL = 1; *P* = 0.58; cervical spine *χ* ^2^ = 0.11; GL = 1; *P* = 0.74; femur *χ* ^2^ = 0.80; GL = 1; *P* = 0.37. As for the BMD evolution compared with the area measured in diabetic males throughout the experiment, significant statistical differences were noticed: overall *χ* ^2^ = 23.42; GL = 1; *P* < 0.001; spine *χ* ^2^ = 11.26; GL = 1; *P* = 0.0008; cervical spine *χ* ^2^ = 9.42; GL = 1; *P* = 0.002; femur *χ* ^2^ = 27.85; GL = 1; *P* < 0.001; the highest being at the femur level. In diabetic males receiving polyphenol treatment, studies showed significant statistical differences in the evolution of their BMD throughout the experiment: overall *χ* ^2^ = 34.60; GL = 1; *P* < 0.001; spine *χ* ^2^ = 56.09; GL = 1; *P* < 0.001; cervical spine *χ* ^2^ = 45.78; GL = 1; *P* < 0.001; femur *χ* ^2^ = 29.07; GL = 1; *P* < 0.001.

Depending on the examined area, the BMC also showed strong statistical differences throughout the surveyed period and in all the bone mineral density tests performed; the highest increase was recorded in the cervical spine, while both femur and overall BMCs were lower at the end than at the beginning of the experiment: overall *χ* ^2^ = 134.81; GL = 1; *P* < 0.001; spine *χ* ^2^ = 809.02; GL = 1; *P* < 0.001; cervical *χ* ^2^ = 1679.20; GL = 1; *P* < 0.001; femur *χ* ^2^ = 52.45; GL = 1; *P* < 0.001.

**Figure 1:**
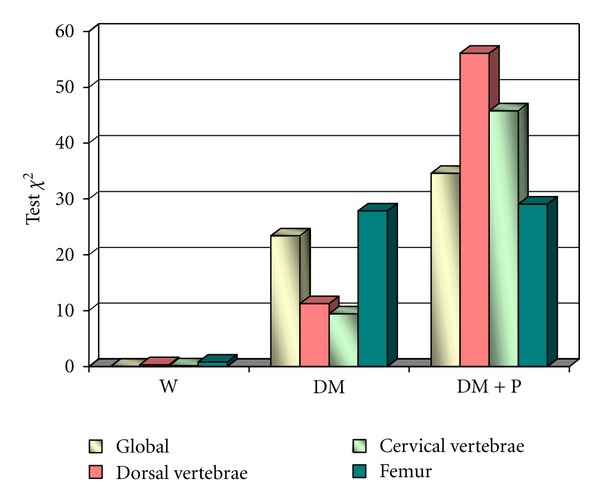
Values of the *χ* ^2^ BMD test for each group.

The comparison between BMC and BMD in diabetic males undergoing polyphenol treatment revealed significant statistical differences both globally and in all the analyzed samples (Figure 1). 

**Figure 2:**
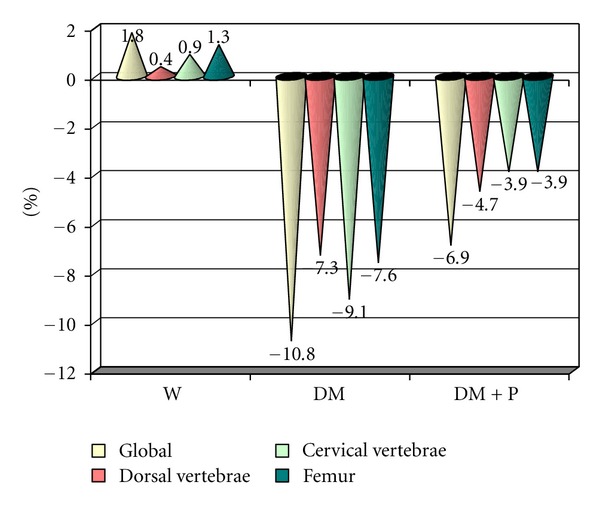
Body fat percentage differences between groups.

The body fat of control males had a slight increase (0.4–1.8%) at the end of the experiment, in all analyzed samples. A 7–11% body fat decrease was noticed in all analyzed samples of diabetic males from the beginning to the end of the experiment. The body fat percentage had the most dramatic decrease in the diabetic male group (Figure 2).

## 4. Discussion

The connection between DM and bone damage associated with mineral metabolism dysfunction is a common phenomenon found in both humans and rats. Insulin is a potential bone growth regulator, since osteoblasts have insulin receptors [9] as well as IGF1 receptors, which can also mediate the effects of insulin. *In vitro* insulin directly stimulates osteoblast proliferation [10] and when administered locally, over the calvariae of adult male mice, it produces two- to three-fold increases in the histomorphometric indices of bone formation [11]. It is also known that insulin promotes amino acid cell intake in cultured bone. Collagen synthesis is increased too, due to a possible direct effect of insulin on the osteoblasts. Insulin also regulates the activity of glycogen synthase in cultured osteoblast-like cells [12].

Some researchers have reported that a longer history of type 1 diabetes is correlated with decreasing bone mass [13, 14]. The mechanisms that lead to a decreased bone biomechanical competence, besides the decreases in BMD, are collagen glycosylation alterations generated by hyperglycaemia, in the same way as increased haemoglobin glycosylation, which is expressed as HbA1C. These advanced glycation end products (AGE) and their receptors (RAGE) play an important role in bone metabolism and bone strength [2].

Our study results are in agreement with the results of other experimental *in vivo* studies conducted on diabetic rats that were treated with vegetal polyphenols found in red wine or green tea [15, 16]. The increased levels of reactive oxygen species (ROS) and decreased antioxidant defense can cause DNA damage and direct protein inhibition. The main substrates targeted by free oxygen radical activity are polyunsaturated fatty acids in membrane phospholipids, the modification of which results in cell framework and function disorganization. The end product of these reactions is MDA. SOD is the first line of defense against ROS and is active in catalyzing superoxide radical detoxification [17]. SOD is the most abundant antioxidant enzyme in animals. A study assessing the systemic oxidant-antioxidant status throughout the evolution of the disease was carried out. SOD decomposes superoxide anion into hydrogen peroxide and oxygen at almost the highest possible reaction rate. The superoxide radical is involved in various physiological and pathophysiological processes. It is produced in respiratory and cytochome P450 electron transport chain reactions as a by-product. A high amount of this product is also generated by activated neutrophils and macrophages during oxidative burst. 

Numerous experimental studies have proven the ability of vegetal polyphenols to diminish lipid peroxidation and to reduce LDL oxidation, probably through the uptake of lipid-peroxil radicals and through lipooxygenases activity reduction, thus delaying atheroma plate formation [18, 19]. Polyphenols are able to penetrate tissues, particularly those in which they are metabolized, but their ability to accumulate in specific target tissues needs to be further investigated. Despite the increasing amount of available data, final conclusions on the bioavailability of most polyphenols are difficult to draw and further studies are necessary [20, 21].

In drug-induced diabetes mellitus with polyphenol protection, the antioxidant quality of the serum increases, the lipid profile is improved and hyperglycemia is significantly ameliorated [22–24]. Two mechanisms were suggested by means of which vegetal polyphenols act as antioxidants within a biological system under oxidative stress conditions [25, 26]: a mechanism according to which polyphenols act as reducing agents or electron donors, converting lipid peroxides in lipid hydroxides or phospholipid peroxides in phospholipid hydroxides, inhibiting lipid peroxidation; another mechanism through which vegetal polyphenols may chelatise transitional metallic ions.

The polyphenolic extract obtained from the *Sambucus nigra* fruit improves the lipid profile significantly (*P* < 0.001) and reduces the atherogenic risk significantly (*P* < 0.001). Hyperglycemia is significantly improved (*P* < 0.01). Aqueous elder (*Sambucus nigra*) extracts have been shown to have an insulin-like effect on *in vitro *glucose uptake [27]. Whereas hyperglycemia can result in the generation of free radicals through several biochemical pathways (nonenzymatic glycation, the polyol pathway, and glucose autoxidation), the mechanisms underlying oxidative damage in diabetes are still unclear. Free radicals can result in antioxidant defense consumption and enhanced susceptibility to lipid peroxidation. The study showed reduced TAS in type 1 diabetic against nondiabetic control subjects. These results indicate that the alterations in the glucose utilizing system and oxidation status in rats increased by streptozotocin were partially reversed by polyphenolic extract delivery.

The TAS reduction in type 1 diabetic rats was associated with increasing HbA_1c_ and diabetes duration. Thus, increased serum levels of the total antioxidant status and HbA_1c_ were associated with osteoporotic risk reduction in diabetic rats.

In diabetic males, the evolution of bone mineral density compared with the area, measured throughout the experiment, suffered a significant decrease, especially in the femur. In diabetic male rats that were given polyphenolic extract, these differences were statistically minimum. 

The highest BMD difference compared with the area was recorded in the spine, followed by the cervical spine. As for the comparison between the BMD of the two groups of males, the most statistically significant difference compared with the area was noticed in diabetic males undergoing polyphenol treatment. 

The BMC in the groups of males had the most statistically significant difference compared with the area in the femur of diabetic males, followed by the cervical spine of males undergoing polyphenol treatment. As for the spine, the highest BMC difference compared with the area was found in diabetic males with polyphenol intake, whereas the highest overall significant difference was noticed in diabetic males. 

Except for the overall comparison, comparisons between the BMC and BMD of diabetic males revealed significant statistical differences in the spine, lumbar, and femur areas. In diabetic males that were given polyphenol treatment, a 4–7% decrease of their body fat was noticed in all analyzed sampled, at the end of the experiment. 

The study revealed reduced TAS in type 1 diabetic against nondiabetic control subjects. The understanding of bone biology may open the way for new osteoporosis treatments. The positive skeletal effects of polyphenols at dietary achievable levels are interleukin-6 mediated [28–30]. 

The experiment shows the benefits of natural polyphenols extracted from the Elder (*Sambucus nigra*) fruit on osteoporosis regression in diabetic male rats. Extremely low bone mineral density in diabetic rats is improved by polyphenol delivery. Significantly low body fat percentages in diabetic rats are also improved by polyphenol intake. By carefully extending these results to humans, one may conclude that a dietary intake rich in natural polyphenols would lead to the regression of diabetes complications in general and osteoporosis in particular, in diabetic human patients. Osteoporosis regression due to the *Sambucus nigra* extract proves the benefits of polyphenols used to treat chronic diabetes mellitus complications.

## Figures and Tables

**Table 1:** The glycaemia evolution at the studied groups.

	Average glycaemia (mg/dL) ± SD		
Group	Week 2	Week 10	Week 16
W	73.20 ± 4.30	72.80 ± 4.10	70.80 ± 3.80
DM	376.55 ±116.82	551.55 ± 166.66	658.44 ± 199.26
DM + P	329.66 ± 112.07	501.66 ± 172.10	529.44 ± 177.74
P	73.40 ± 4.40	71.80 ± 3.90	70.60 ± 3.70

**Table 2:** TAS, blood GPX activity, SOD activity, and IL-6 values in the studied group.

Experimental groups	W	P	DM	DM + P
TAS (mmoli/L)	1.2148 ± 0.0122	1.3313 ± 0.0339*	1.0464 ± 0.0526***	1.308 ± 0.0224^##^
GPX (nmol/mL)	7.60 ± 0.20	7.84 ± 0.46*	5.16 ± 0.54***	6.84 ± 0.62^##^
SOD (U/mL)	3.60 ± 0.38	4.92 ± 0.60***	2.86 ± 0.24*	3.28 ± 0.18^#^
MDA (*μ*moli/mL)	0.068 ± 0.002	0.064 ± 0.004*	1.213 ± 0.072***	0.847 ± 0.36^##^
IL-6 (ng/mL)	8.35 ± 0.323	8.52 ± 0.32*	27.19 ± 3.482***	13.90 ± 3.013^###^

Results are mean ± SEM (*n* = 12).

*P < 0.05, **P < 0.01  ***P < 0.001 versus W group.

^#^ *P* < 0.05, ^##^ *P* < 0.01, ^###^ *P* < 0.001 versus DM group.
